# Oral health knowledge, attitude and practices among student nurses at a university in South Africa

**DOI:** 10.4102/hsag.v31i0.3516

**Published:** 2026-10-22

**Authors:** Pelisa Khonco, John J. Musafiri, Jennifer-Anne Chipps

**Affiliations:** 1 School of Nursing, Faculty of Community and Health Sciences, University of the Western Cape, Cape Town, South Africa

**Keywords:** oral health, student nurses, attitudes, knowledge, oral practices

## Abstract

**Background:**

Poor oral health resulting from poor knowledge, negative attitudes and poor practices contributes to oral diseases. Oral diseases remain a public health concern and negatively affect student nurses’ academic performance. These students should serve as role models in promoting oral health among communities. A lack of oral self-care among student nurses may raise a health concern.

**Aim:**

To assess student nurses’ knowledge, determine their attitude and practices regarding oral health at a Western Cape university.

**Setting:**

This study was conducted at a Western Cape university, South Africa, which offers undergraduate nursing programmes.

**Methods:**

A quantitative approach with a descriptive design was conducted, and data were collected from a quota sample of 287 student nurses using a self-administered questionnaire. Data captured into IBM Statistical Package for the Social Sciences computer program IBM SPSS Statistics version 28.0 (IBM Corp., Armonk, NY, United States), were analysed using descriptive statistics and Chi-square tests to test associations.

**Results:**

The response rate was 97.9% (281/287). Most of the respondents did not recognise the importance of brushing twice daily and flossing once, and only 98 (34.9%) were knowledgeable. A positive attitude was noted among the majority of the respondents (261, 92.9%) who agreed that oral health knowledge can contribute to the prevention of tooth loss. Good practices were noted among most of the respondents (277, 98.6%) who cleaned their teeth daily with a toothbrush.

**Conclusion:**

Student nurses had poor oral health knowledge, mixed attitudes and fair oral health practices. This study highlights the need for the formal integration of oral health into the nursing curriculum.

**Contribution:**

This study contributed to the body of knowledge in nursing research and education.

## Introduction

Oral diseases such as dental caries, periodontal diseases, tooth loss and oral cancers constitute a public health concern and affect approximately 3.5 billion people worldwide (World Health Organization [WHO] 2025). Two billion people across the world are estimated to suffer from dental caries of permanent teeth (WHO 2020). Factors such as poor nutrition, alcoholism, smoking, a lack of oral health knowledge, oral health negative attitudes and poor practices contribute to the development of oral diseases (Tadin et al. 2022). Common oral diseases such as tooth decay, dental carries and periodontal disease are chronic oral diseases that can lead to systemic diseases such as diabetes mellitus and cardiovascular disease (Ahmed et al. 2023; WHO 2020). In South Africa, there is great variability in oral health status with a high prevalence of poor oral health status in underprivileged communities due to the unequal distribution of dental facilities and dentists (Mbele-Kokela & Moodley 2021).

University students, like the public, can suffer from oral diseases (Corrêa et al. 2023; Elwadia et al. 2024; Mbele-Kokela & Moodley 2021). Student lifestyles such as smoking, consumption of alcohol and sugar-containing drinks and unhealthy food intake can increase the risks of developing oral diseases (Azam et al. 2024). Oral diseases can contribute to poor academic performance, increase dropout, reduce students’ participation in social activities, decrease their self-esteem and cause physical and psychological symptoms (Alsaç et al. 2024; Corrêa et al. 2023; Elwadia et al. 2024; Gardner, Huang & Ip 2023). A study conducted among university students in Qatar showed that 72.3% of the 112 respondents had interrupted sleep due to oral problems, 84.8% were taking days off class and 85.7% were not able to carry out daily activities (Elwadia et al. 2024).

Globally, studies conducted on university students in Croatia, Qatar and Bangladesh showed that the majority of the respondents had good oral health knowledge (Elwadia et al. 2024; Islam et al. 2025; Tadin et al. 2022). However, it is essential that student nurses, who are responsible for promoting oral health, have good oral health knowledge (Lundell Rudberg et al. 2022). There are reports, of inconsistency in the emphasis of oral health in nursing curricula (Grønkjær et al. 2017), which can result in inadequate oral health knowledge, negative attitudes and poor practices among student nurses (Yavagal et al. 2020). In a study carried out in Denmark, senior students nurses acceptable oral health knowledge in some areas, although their knowledge of periodontal diseases (28%) out of 100 was insufficient. the majority of students (87%) recognised the importance of oral health for general health (Grønkjær et al., 2017). A study conducted with student nurses in India, showed good knowledge of oral health care and high awareness of oral diseases risk factors to systemic diseases. Nearly half of the respondents visited dentists only when they had a dental problem, indicating a lack of knowledge (Yavagal et al. 2020), confirming that nurses lack confidence in oral care because they lack knowledge (Haresaku et al. 2020). In KwaZulu-Natal province, South Africa, more than half of student nurses perceived their oral health as good or excellent irrespective of their year of study, but 17% reported gum bleeding during flossing and 8% reported halitosis, indicating some students have experienced oral health problems (Singh & Kerr 2018). This discrepancy between perceived and actual oral health may suggest gaps in the oral health knowledge. Given the important role nurses play in health promotion, poor oral health among student nurses may affect their ability and willingness to effectively integrate oral health assessment, education and preventive practices into patient care.

The variability in global and local findings highlighted the need to investigate the oral health status of student nurses as student nurses’ oral health practices play an essential role in providing oral health education to their patients (Yavagal et al. 2020). Oral health self-care is fundamental practice to promote general well-being (Onwubu et al. 2022), and it is recommended to improve attitudes on oral health care and to integrate formal oral health education in the nursing curriculum (Bhagat et al. 2020).

There is a limited number of studies that examined oral health knowledge, attitudes, and practices among student nurses (Cabov et al. 2022; Nazarianpirdosti et al. 2021). The availability of only one dated South African study on this population (Singh & Kerr 2018) and the predominance of research focusing on general university students rather than nursing students specifically (Barahona-Cubillo, Rojas-Brenes & Barboza-Solís 2024; Islam et al. 2025). The current study aimed to address this evidence gap by investigating the oral health status, knowledge, attitudes and practices of student nurses.

### Study aim

The study aim was to evaluate the oral health status and the knowledge, attitudes and practices of student nurses’ regarding oral health at a Western Cape University.

## Research methods and design

A quantitative research approach with a descriptive design using a self-administered questionnaire was used to collect data related to oral knowledge, attitudes and practices among student nurses.

### Setting

The study was implemented at a Western Cape University where student nurses were enrolled in 2020 in various programmes including the Bachelor of Nursing programme. The student nurses attend theory classes and are placed in healthcare facilities for clinical learning. These students care for the patients who might have oral care problems and need oral self-care education.

### Study population and sample

The population included 1098 student nurses enrolled at a Western Cape University in a nursing programme at the time of data collection. The sample size was calculated using the formula (population size = 1098, distribution *p* = 50%, margin of error = 5% and confidence level = 95%) with a recommended sample size of 287. Quota sampling was used to ensure proportional distribution across the year levels was applied.

### Instruments

A self-administered questionnaire was developed and included two socio-demographic questions and three validated scales, namely the ‘Oral Health Questionnaire for Adults’ (WHO 2013), the ‘Rustvold Oral Health Knowledge Inventory’ (Rustvold 2012) and questions related to student nurses’ attitudes towards patients’ oral care (Andargie & Kassahun 2019). The Oral Health Questionnaire had 42 *Yes, No* questions on the oral health of student nurses (WHO 2013) (*α* = 0.755 in this study for problems with teeth). The Rustvold Oral Health Knowledge Inventory had 10 closed, multiple-choice questions to test student nurse’s knowledge on oral health. No internal consistency was assessed due to the short scale for knowledge with just 10 multiple choice questions each measuring different constructs of knowledge. The Oral Health Attitudes Questionnaire had nine negative statements (*α* = 0.619 in this study) and five positive statements measuring student nurse’s attitude towards oral health (Rustvold 2012) (*α* = 0.606 in this study). A pre-test of the questionnaire with 12 student nurses (three per year level) was conducted to test its reliability in a South African context. No changes were required to the questionnaire items post pre-test. The 12 pre-test respondents’ data were included in the final sample.

### Data collection

Student nurses were contacted via email and invited to participate in the study. The potential participants were told about the data collection venue, date and time. Collection was done after class, and data were collected by the researcher and assisted by non-teaching staff members. Questionnaire completion took approximately 30 min on average, and data collection was conducted over a 4-week period.

### Data analysis

The data were captured, cleaned and analysed in the Statistical Package for the Social Sciences (SPSS) software version 28. Descriptive statistics using frequencies, percentages and means were employed to describe data related to demographic characteristics, oral health status, knowledge, attitudes and practices. Poor oral health status was defined as any respondent that had < 20 teeth or complained of pain in the last 12 months or rated their teeth or gums in poor health. Knowledge was tested with 10 questions related to oral health knowledge, and answers were marked as correct or incorrect with a total mark of 10. Respondents who scored < 50% and below were classified as not being knowledgeable, between 50% and 80% as somewhat knowledgeable and above 80% as knowledgeable (Kamacooko et al. 2021). The practice scale data consisted of the Likert scale, and the frequency of the practice was coded into two categories, namely: *Often* and *Seldom*. The attitude scale data were coded with ‘*I disagree partly*, and *I disagree*’ coded as ‘*Disagreed*’. A Chi-square test was used to examine the association between respondents’ demographic characteristics to their oral health knowledge, attitudes and practices. Means and frequencies were calculated, and associations of demographics with knowledge, attitudes and practices were tested using nonparametric statistics as appropriate.

### Ethical considerations

Ethical clearance to conduct this study was obtained from the Humanities and Social Science Research Ethics Committee of the University of the Western Cape (No. HS20/8/6). Permission to access the nursing students was obtained from the Registrar of the University and the Head of Nursing Department. All three research fundamental principles were applied. The respondents’ rights were considered, and the consenting respondents completed the questionnaires.

## Results

### Demographics and sample realisation

The response rate was 281/287(97.9%), with most respondents being female (235, 83.6%). The average age was 21.9 ± 3.5 years, with ages ranging from 18 to 38 years. A greater proportion of respondents was single (245, 87.2%). Most of the respondents were enrolled in the first-year level of study (81, 28.8%), followed by the fourth-year level students (79, 28.1) (Table 1).

**TABLE 1:** Respondents’ demographic characteristics (*N* = 281).

Content	*n*	%
**Gender**		
Male	46	16.4
Female	235	83.6
**Year level of study**		
1st	81	28.8
2nd	75	26.7
3rd	46	16.4
4th	79	28.1

*Source:* Khonco, P., 2022, *Knowledge, attitudes and practices of student nurses regarding oral health at a university in the Western Cape*, University of the Western Cape, viewed 14 July 2026, from https://hdl.handle.net/10566/19035

### Respondents’ oral health status

The respondents were asked to describe their teeth and mouth condition, as well as any related difficulties experienced in the previous year (Table 2). Nearly all respondents had more than 20 teeth (264, 94%), with only 25 (8.9%) having a dental prosthesis. Nearly two-thirds (176, 62.6%) of the respondents had experienced no dental problems in the previous year with 208 (74.0%) and 213 (75.5%) rating their teeth and gums in excellent condition (Table 2).

**TABLE 2:** Respondents’ oral health status (*N* = 281).

Content	*n*	%
**Number of teeth**		
Own teeth (≥ 20)	264	94.0
Own teeth (0–19)	17	6.0
**Dental problems**		
Absence of dental problems in the previous year	176	62.6
Dental problems in the previous year	99	35.2
Uncertain	4	1.4
**Dental prosthesis**		
A partial dental prosthesis	17	6.0
A full upper dental prosthesis	3	1.1
A full lower dental prosthesis	5	1.8
**Teeth condition**		
Good to excellent teeth condition	208	74.0
Average teeth condition	66	23.5
Poor teeth condition	7	2.5
**Condition of gums**		
Good to excellent condition of gums	213	75.8
Average condition of gums	57	20.3
Poor condition of gums	11	4.0

*Source*: Khonco, P., 2022, *Knowledge, attitudes and practices of student nurses regarding oral health at a university in the Western Cape*, University of the Western Cape, viewed 14 July 2026, from https://hdl.handle.net/10566/19035

Nearly half of the respondents (118, 42%) were classified as having poor oral health status, that is, any respondent that had < 20 teeth or complained of pain in the past year or rated their teeth or gum condition in poor health. There were no significant differences in year level (*X*^2^ = 1.5, *p* = 0.682).

### Respondents’ oral health knowledge

The respondents were asked 10 questions to measure different constructs related to oral health knowledge. The respondents’ average score was 4.6/10 ± 1.7 (47.8%) with 136, (48.4%) scoring below 50% and were misinformed, 129 (45.9%) scored between 50% and 80% (somewhat knowledgeable) and only 16 (5.7%) scored above 80% and were classified as knowledgeable. Female respondents had statistically higher knowledge scores (4.7/10 ± 1.7 vs 4.02/10 ± 1.5 *U* = 2.6, *p* = 0.009), although still below 5/10. Respondents in their first year of study scored lower than the other years although still below 5/10, and significantly lower than third years (Year 1 – 4.21/10, ± 1.60, Year 2 – 4.59/10, ± 1.69, Year 3 – 5.35/10, ± 1.84 and Year 4 – 4.53/10, ± 1.76, *K* = 11.6, *p* = 0.009). There was no significant difference in knowledge between respondents who visited the dentist in the last year or not (*U* = 1.6 *p* = 0.124).

Assessing the individual knowledge questions, the question related to gingivitis was correctly answered by the highest proportion of respondents (238, 84.6%). Most of the respondents answered correctly one question related to the definition of plaque (208, 74.0%), another related to less effect of sugar consumption on the teeth (213, 75.9%). However, only the minority (81, 28.2%) could recognise the association between oral diseases and general health (Table 3). Analysis by year of study revealed significant differences in oral health knowledge. First-year respondents were significantly less likely to correctly identify gingivitis than respondents were in the second, third and fourth years (66.7% vs. 89.3%, 93.5% and 92.4%, respectively; *χ*^2^ = 27.3, *p* < 0.001). Similarly, third-year respondents had demonstrated significantly higher knowledge regarding the purpose of tooth brushing (*χ*^2^ = 10.4, *p* = 0.017), the link between periodontal disease and general health (*χ*^2^ = 12.8, *p* = 0.005), and the link between periodontal disease and cigarette smoking (*χ*^2^ = 12.9, *p* = 0.005) than respondents in the other year groups.

**TABLE 3:** Respondents’ oral health knowledge (*N* = 281).

Content	Correct answer		Incorrect answer	
	*n*	%	*n*	%
Gum inflammation that is characterised by swelling and bleeding.†	238	84.6	43	15.4
The best time to consume sugary snacks is after a meal.	213	75.9	68	24.1
Plaque – a bacteria-containing substance found on the surface of teeth.	208	74.0	73	26.0
Toothpaste containing fluoride has noticeably improved oral health by preventing dental cavities.	181	64.4	100	35.6
Cigarette smoking contributes to periodontal disease cases and various oral cancers.†	153	54.4	123	45.6
Bacteria have the ability to change sugar into acid that is harmful to the tooth enamel.	113	40.2	163	59.8
Daily flossing is an essential part of your dental health routine, and minor gum bleeding at the beginning is normal.	105	37.4	176	62.6
The normal daily dental care routine includes brushing twice a day and flossing once.	98	34.9	183	65.1
The aim of brushing teeth to remove microbes, and food particles.†	97	34.5	184	65.5
Periodontal diseases and oral health connection may affect general health, e.g. premature birth, diabetes mellitus and cardiac diseases.†	81	28.2	200	71.8

*Source*: Khonco, P., 2022, *Knowledge, attitudes and practices of student nurses regarding oral health at a university in the Western Cape*, University of the Western Cape, viewed 14 July 2026, from https://hdl.handle.net/10566/19035

† , Significant higher proportion of third-year students providing the correct answer.

### Respondents’ oral health attitudes

Respondents had to rate the level of agreement on five statements related to positive oral health attitudes and nine statements evaluating respondents’ perceived barriers to maintaining optimal oral health.

Positive oral health attitudes had a high level of agreement with all statements having > 80% level of agreement. Most of the respondents (261, 92.9%) agreed that *knowledge of oral health facts is a factor contributing to prevention of tooth loss*, and this statement had the highest level of agreement followed by agreement *I believe I am using the correct technique in brushing my teeth* (257, 91.5%). There were no significant differences between respondents’ gender or year level for the positive statements.

The respondents were asked to indicate their perception about the barriers to optimum oral health. The highest level of agreement was for *I believe if my gums bleed during flossing it is an indication that I should stop flossing as I am harming my gums* with 199 (70.8%) agreeing with this statement, followed by *I believe that losing teeth is a normal part of growing old* (178, 63.3%) (Table 4). Overall, respondents had low levels of agreement with the proposed barriers to achieve optimal oral health (< 50%).

**TABLE 4:** Respondents’ oral health attitude (*N* = 281).

Content	Agreement	
	*n*	%
**Optimum oral health statements**		
If I had adequate knowledge about oral health, I could help reduce tooth loss.	261	92.9
I believe I am using the correct technique in brushing my teeth.	257	91.5
I believe regular flossing of my teeth is a preventative measure for gingivitis.	239	85.1
I believe it is my responsibility to ensure that I do not lose my teeth.	234	83.3
I believe that consistent tooth brushing and flossing decrease the susceptibility of tooth loss.	230	81.9
**Barriers to optimum oral health statements**		
I believe if my gums bleed during flossing it is an indication that I should stop flossing as I am harming my gums.	199	70.8
I believe that losing teeth is a normal part of growing old.	178	63.3
I believe if my gums bleed during brushing it is an indication that I should stop brushing as I am brushing my teeth hard.	120	42.7
I believe only dentists are able to prevent tooth decay.	87	31.0
I believe it is easier to take care of dental prosthesis than it is to care for my own teeth.	78	27.8
I believe dentist visits should only be done when I am experiencing pain.	78	27.8
I believe I am at risk of developing tooth caries in the next year or two.	58	20.6
I believe I am at risk of developing gingivitis or gum disease in the next year or two.	48	17.1
I believe that having parents with dental problems brushing and flossing will not benefit my teeth.	30	10.7

*Source*: Khonco, P., 2022, *Knowledge, attitudes and practices of student nurses regarding oral health at a university in the Western Cape*, University of the Western Cape, viewed 14 July 2026, from https://hdl.handle.net/10566/19035

However, significantly more female respondents (131, 55.7%) compared to (15, 32.6%) male respondents believed that *dental prostheses are easier to maintain than natural teeth* (*X*^2^ = 10.2, *p* = 0.017), and more female respondents (113, 48.1%) compared to 18 (39.16%) male respondents believed that dental check-ups are only necessary when experiencing pain (*X*^2^ = 11.8, *p* = 0.008) (Table 4). Significant differences in beliefs were observed across the different years of study, with a significantly higher proportion of first-year respondents believed that *losing teeth is a normal part of growing old* (Khonco 2022), compared to second, third and fourth year respondents (61, 75.3% vs 49, 65.3% (Second Year), 27, 58.7% (Third Year) and 41, 15.9% (Fourth Year) (*X*^2^ = 10.0, *p* = 0.019).

### Respondents’ oral health practices

Oral practices were examined through questions on mouth hygiene, visits to the dentist and usual food habits, which may have an impact on their oral health. Nearly two-thirds of the respondents cleaned their mouth twice daily were 171 (60.9%), while only 89 (31.7%) cleaned it once a day and 201(71.5%) used a toothpaste with fluoride. Overall, 92 (32.7%) respondents reported having visited a dentist during the previous year, while nearly one-quarter, that is, 66 (23.5%) had never visited a dentist. Analysis showed no significant differences between males and females in dental attendance during the past year (*X*^2^ = 1.0, *p* = 0.313) or among year levels *(X*^2^ = 8.9, *p* = 0.115). Reasons reported as to why respondents visited the dentist were for toothache (78, 36.3%), routine check-ups (73, 34%), treatment follow-up (22, 10.2%) and consultation (22, 10.2%) (Table 5).

**TABLE 5:** Respondents’ oral health practices (*N* = 281).

Content	Yes	
	*n*	%
**Best hygiene practices**		
Clean twice a day	171	60.9
Clean once a day	89	31.7
**Poor hygiene practices**		
Clean 2–6 times a week	21	7.4
**What do you clean your teeth with**		
Used toothbrush to clean	277	98.6
Used toothpicks (wood)	137	48.8
Used thread (floss)	135	48.0
Do you use toothpaste with fluoride	201	71.5
**When was your last dentist visit**		
Visited the dentist in last year	92	32.7
Visited dentist more than a year ago	158	43.8
Never visited the dentist	66	23.5
**What was the reason for your dentist visit (*N* = 215)**		
Pain	78	36.3
Routine check-up	73	34.0
Consultation	24	11.2
Treatment follow-up	22	10.2
Uncertain	18	8.4

*Source*: Khonco, P., 2022, *Knowledge, attitudes and practices of student nurses regarding oral health at a university in the Western Cape*, University of the Western Cape, viewed 14 July 2026, from https://hdl.handle.net/10566/19035

### Food habits and substances that may harm teeth

Respondents were asked questions related to the intake of foods and substances that may affect the health of their teeth and gums. Over two-thirds (195, 69.4%) indicated that they ate fresh fruit often; fresh fruit contains nutrients and vitamins that the body may need; however, high fruit intake may harm teeth enamel due to the natural sugars and acids found in fruits and juices made with fresh fruit (Inchingolo et al. 2023). Nearly two-thirds (177, 63%) often drank tea or coffee with sugar (significant differences between gender [male 26{78.8%} vs. female 152 {92.1%}, *X*^2^ = 5.3, *p* = 0.021] and no significant differences between year levels [*X*^2^ = 5.3, *p* = 0.377]). Just over 28 (10%) reported using cigarettes frequently (Table 6).

**TABLE 6:** Food and substance often consumed in small quantities (*N* = 281).

Content	Often (daily or several times a week)	
	*n*	%
**Food consumed**		
Eat fresh fruit	195	69.4
Tea or coffeee with sugar	177	63.0
Sugar-containing soft drinks such as lemonade and Coca-Cola	124	44.1
Eat sweets or candy, biscuits, cakes, sweet buns, sweet pies or cream cakes, chew gum with sugar, jam or honey	242	86.1
**Substances used**		
Used cigarettes	28	10.0
Other tobacco substances	10	3.6

*Source:* Khonco, P., 2022, *Knowledge, attitudes and practices of student nurses regarding oral health at a university in the Western Cape*, University of the Western Cape, viewed 14 July 2026, from https://hdl.handle.net/10566/19035

## Discussion

As nurses are key role players in educating patients on oral health, this study aimed to investigate the oral health status, knowledge, attitudes and practices of student nurses from a Western Cape university. Overall, the findings indicate that nearly half of the respondents reported poor oral health status, had inadequate oral health knowledge, variable oral health practices but generally positive attitudes towards oral health, suggesting a disconnect between attitudes and behaviour.

### Oral health status

Poor oral health among university students can contribute to their poor academic performance and absenteeism in class (Corrêa et al. 2023; Karam et al. 2021). In this study, nearly half of the respondents were classified as having poor oral health status, with no significant differences in year levels. Most of the respondents had their own teeth (94.0%) but only three quarters rated their teeth and gums to be in a good state, and a third reported pain or discomfort in the past 12 months. These findings seem in line with other studies such as a study in Denmark where just over half of the student nurses indicated that they had toothache and mouth pain in the past 12 months (Grønkjær et al. 2017) and 20% described their teeth and gums as not in a good state (Grønkjær et al. 2017). Ensuring gum health is important as gums support teeth and aid in preventing bad breath and may decrease pain and discomfort (Sharma 2023). The findings of this study support the possible impact of poor oral health knowledge and practices and highlight a further concern regarding their role as future healthcare professionals who need to be adequately equipped with oral health knowledge to promote oral health among their patients.

### Respondents’ oral health knowledge

As indicated, a major concern was the poor level of oral health knowledge, with more than half failing the knowledge assessment across several oral health topics and significant differences in knowledge between male and female respondents. Female respondents had significantly higher oral health knowledge scores than male respondents, although both scores were below 50%. These findings are like those of a study conducted in Saudi Arabia where oral health knowledge scores were significantly higher among females than males (8.5 ± 1.5 vs 8.1 ± 1.7) (Rajeh 2022) and in India, where higher oral health knowledge scores were reported among female student nurse respondents (*p* < 0.001) than male respondents (Lakshmi et al. 2022). The fact that females are more likely to engage in self-care than males can be associated with their willingness to look for information to take care of their oral health (Sfeatcu et al. 2022).

Of particular concern among the overall group was the limited understanding of the relationship between periodontal disease and general health, with nearly three-quarters of respondents unaware that periodontal disease can have systemic health implications. This finding is consistent with a study conducted among undergraduate nursing students in India, where approximately three-quarters of respondents were similarly unaware of the association between oral diseases and systemic conditions (Verma & Dhruvakumar 2021). Adequate knowledge of the interrelationship between oral and general health is essential to enable them to effectively educate patients and communities, identify risk factors and incorporate oral health promotion into routine nursing care.

A second concern was the inconsistent knowledge and practices related to oral health risk factors. Although respondents had the highest scores for knowledge of gingivitis and dental plaque, only slightly more than half were able to identify the risk factors for oral diseases. This was lower than a study conducted in Spain which found that approximately 80% of nursing students correctly identified the risk factors associated with gingivitis (Marquès-Pellejà et al. 2023). In contrast the respondents indicated reasonable knowledge with nearly two-thirds of the respondents knowing the importance of the use of fluoride, knowledge which is essential for reducing dental caries (Vorster & Naidoo 2019) and three quarters using fluoride when brushing their teeth, similar to a study done in Pakistan (Bashir et al. 2021). However, there was a lack of knowledge about the frequency and purpose of brushing and flossing teeth with three quarters not knowing that the aim of brushing teeth, and only 34.9% had knowledge of the recommended frequency of brushing and flossing, which can contribute to poor health teeth practice among student nurses who should be role models in the community. Similar findings were also documented among student nurses in India where only 3% were knowledgeable about brushing twice a day (Yavagal et al. 2020). These findings also concur with those of a study conducted in China where undergraduate student nurses had poor knowledge of interdental cleaning with 50.5% of undergraduate student nurses never flossing their teeth (Chan & Chin 2017).

Lastly, a substantial proportion of respondents lacked knowledge regarding the association between smoking and oral diseases, which was like research among nursing students in India reported deficiencies in knowledge of risk factors for oral cancer (Kaliamoorthy, Dhamodharan & Caliaperoumal 2021). Given the well-established link between tobacco use and an increased risk of periodontal disease, oral cancer and other oral health conditions (Fellows et al. 2022), this knowledge gap should be addressed.

The differences observed on oral health knowledge, practices and knowledge of risk factors for periodontal diseases across the year levels may be influenced by the nursing curriculum variations. Within the regulation 425 (R425) curriculum, student nurses were introduced to the basics of oral health primarily in the general nursing science at second-year level, and the oral health knowledge was consolidated in the midwifery module at the third-year level. As a result, the third-year student nurse respondents had higher knowledge scores than the second-year respondents, and first-year level student respondents had significantly lower scores. However, it is noted that the fourth-year student nurse respondents had lower knowledge scores than third-year respondents probably due to the focus on psychiatric nursing across the academic year at the fourth-year level. This is a major concern as a review found that people with mental health problems consistently demonstrate a high prevalence of poor oral health outcomes (caries, tooth loss and periodontal disease) and significantly lower engagement in oral hygiene (for example, brushing teeth) and dental care-seeking behaviours (Afroz et al. 2025). In contrast, a study in India reported higher oral health knowledge scores among fourth-year student nurses (Yavagal et al. 2020). Furthermore, studies in India showed that mean oral health knowledge scores were significantly higher among the third- and fourth-year student nurses than the first- and second-year students (Verma & Dhruvakumar, 2021; Yavagal et al. 2020).

These finding suggests that while student nurses possessed basic knowledge of common oral health conditions, their knowledge of oral health-related disease prevention and oral health risk was less developed. These findings are consistent with those reported in other studies and may represent a broader challenge among nursing students internationally. Given the central role of nurses in health promotion and disease prevention, the ability to recognise and communicate oral disease risk factors is essential for effective patient education and early intervention.

The gap in oral health knowledge among student nurses raises a concern, given that these students are expected to educate the community and their basic oral health knowledge to transfer to the community might be limited. These findings highlight gaps in the oral health knowledge and highlight the importance of incorporating oral health knowledge from the first year in the nursing curriculum. A study conducted on student nurses in Spain recommended that the undergraduate nursing curriculum should improve the content on oral health (Marquès-Pellejà et al. 2023), and based on our findings, this is also a recommendation from our study.

### Respondents’ oral health attitudes

The respondents demonstrated positive attitudes towards their basic dental care with high levels of agreement among respondents regarding personal oral care. Nearly all of the respondents believed that being knowledgeable about oral health could avoid loss of their teeth and expressed confidence that they used correct teeth brushing techniques. Similarly, most (80%) of the respondents believed that consistent brushing of teeth and regular flossing is a preventative measure for gingivitis and subsequently reducing tooth loss. Similarly, in Nepal, most of the student nurse respondents believed that oral knowledge could prevent tooth loss and that they were using correct brushing techniques (Bhattarai et al. 2016). Likewise, most of the respondents (147; 80%) among student nurses who participated in a study undertaken in Jordan considered good oral hygiene important and preventative of oral diseases (Smadi & Nassar 2016), and in Denmark, nearly all the respondents considered oral hygiene an important component for nursing and general well-being (Grønkjær et al. 2017).

Although no significant differences were observed in year level and gender, more female respondents believed that dentures are less trouble than taking care of natural teeth, and visiting the dentist is only necessary when experiencing pain. In other studies, significant differences between males and female respondents were observed with female students demonstrating different attitudes than male student nurses (Lakshmi et al. 2022). In Spain, having a family member working in a healthcare facility was the only student nurses’ socio-demographic factor that significantly influenced their oral health attitudes (Marquès-Pellejà et al. 2023).

### Respondents’ oral health practices

Practices in this study were fair. Although most practices were not different between year level and gender, significantly more females reported drinking tea/coffee with sugar more than males. This behaviour is of concern as added sugar is a major contributing factor to dental caries (Valenzuela et al. 2021). Although comparative evidence in nursing students is limited, it is consistent with evidence that females tend to have a higher sugar intake than males (Hallam et al. 2016), and that this can be related to more dental caries where female students in China reported more dental caries than male students (24.1%) (Chen et al. 2025).

The majority of the respondents reported brushing their teeth using a toothbrush with a fluoridated toothpaste. However, under half of the respondents reported interdentally cleaning using wooded toothpicks and thread. Dental visits were poor, possibly related to access, with around a quarter of respondents only visiting due to pain and some reported having never visited a dentist. The findings are aligned with those of a study conducted in India in 2019 on student nurses where only a third of dental visits were due to oral discomfort (Yadav et al. 2019). Similarly, the findings of a study conducted in India on student nurses indicated that 6% never visited a dentist and similarly under half of the respondents visited the dentist when there was a problem (Yavagal et al. 2020).

### Limitations

The limitations of this study should be acknowledged. The assessment of oral health knowledge was based on a limited number of multiple-choice questions and may therefore not have fully reflected respondents’ overall level of oral health knowledge. Future studies should consider using more comprehensive instruments to assess different domains of oral health knowledge. In addition, the study sample was drawn from a single nursing school within one South African province, limiting the extent to which the findings can be generalised to other nursing student populations. Furthermore, the inability to conduct random stratified sampling, owing to restricted access to class lists under *Protection of Personal Information Act (POPIA)* regulations (Adams et al. 2021), may have introduced selection bias and affected the representativeness of the sample.

### Recommendations

Nursing education should expand the amount of oral health content in their curriculum, especially with the first-year level students as they scored the lowest on some oral health knowledge items than the rest of the year levels and in fourth-year students as dental health problems are a major issue in mental health patients. This approach would be beneficial for the general health quality of patients and for enhancing the oral health knowledge of student nurses. Student nurses can receive health education through clinical skills, workshops and oral health campaigns. Including oral health information in the nursing curriculum and through hosting oral health campaigns for student nurses will encourage them to adopt good oral health and general health practices and empower student nurses to champion the oral and general health of their patients. It is recommended that future researchers conduct a qualitative study to investigate oral health awareness among student nurses.

## Conclusion

This study’s findings show poor knowledge, mixed attitude and fair practices regarding oral health among student nurses. The nursing schools should enhance oral health awareness among student nurses.
